# Conversion of Geraniol into Useful Value-Added Products in the Presence of Catalysts of Natural Origin: Diatomite and Alum

**DOI:** 10.3390/ma15072449

**Published:** 2022-03-26

**Authors:** Anna Fajdek-Bieda, Agnieszka Wróblewska, Piotr Miądlicki, Anna Konstanciak

**Affiliations:** 1 Technical Department, Jacob of Paradies University, Chopina 52, 66-400 Gorzow Wielkopolski, Poland; abieda@ajp.edu.pl; 2 Department of Catalytic and Sorbent Materials Engineering, Faculty of Chemical Technology and Engineering, West Pomeranian University of Technology in Szczecin, Piastów Ave. 42, 71-065 Szczecin, Poland; piotr.miadlicki@zut.edu.pl

**Keywords:** diatomite, alum, geraniol, beta-pinene, 6,11-dimethyl-2,6,10-dodecatriene-1-ol, thumbergol

## Abstract

This article presents research related to the transformation of geraniol (GA), leading to the formation of products with very valuable properties. In the planned method, heterogeneous catalysts of natural origin in the form of alum and diatomite were used as catalysts. Initially, the process which we investigated was the process of isomerization of GA, but it turned out during the studies that GA is also transformed in other reactions. Before catalytic tests, these two minerals were subjected to detailed instrumental analyses using the following methods: XRD, SEM/EDX, XRF and FTIR, which allowed to obtain their full morphological characteristics. During the catalytic tests, the influence of such relevant parameters on the GA transformations was determined: temperature from 80 to 150 °C, catalyst content from 5 to 15% by weight and the reaction time from 15 min to 24 h. The tests presented in the article were carried out under atmospheric pressure (in air) as well as without the use of a solvent. The optimal conditions for the transformations of GA were determined on the basis of its conversion and selectivities of transformation to the main products in the form of: beta-pinene (BP), 6,11-dimethyl-2,6,10-dodecatriene-1-ol (DC) and thumbergol (TH). The above products were formed with the highest selectivity, respectively: 100 mol%, 50 mol% and 52 mol%. The results of the syntheses showed that for GA the best transformation results were obtained at the temperature of 80 °C (for both tested catalysts), with the catalyst content of 1 wt % (for both tested catalysts) and for the reaction time of 1 h (for diatomite)) and 3 h (for alum).

## 1. Introduction

**Figure 1:**
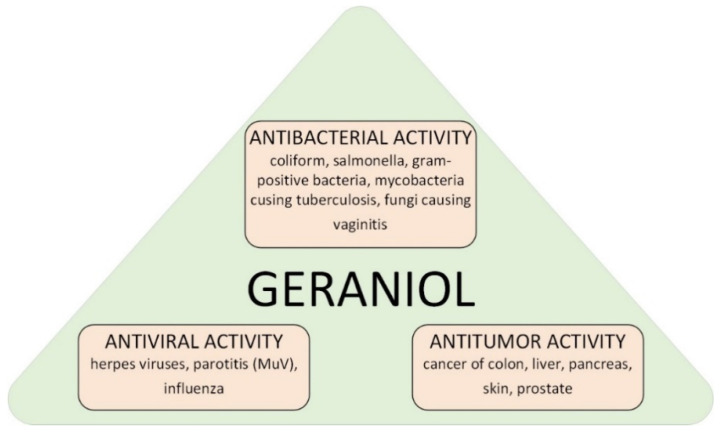
Possible ways of applications of geraniol in medicine.

Geraniol (GA) (C_10_H_18_O, (2*E*)-3,7-dimethylocta-2,6-dien-1-ol) is a chemical compound from the group of monoterpene alcohols. It is a commonly used fragrance ingredient in perfumes, creams, powders, shampoos, and toilet soaps [1,2,3]. On an industrial scale GA is isolated from such plants as *Pelargonium graveolens* or *Cymbopogon winterianus* [4,5,6]. GA shows a variety of biological effects (Figure 1) [7,8,9,10]. Literature reports confirmed its effective action in the treatment of intestinal, skin, liver, kidney and prostate cancer [11,12,13,14]. GA also has anti-inflammatory, antibacterial and antioxidant properties [15,16].

**Figure 2:**
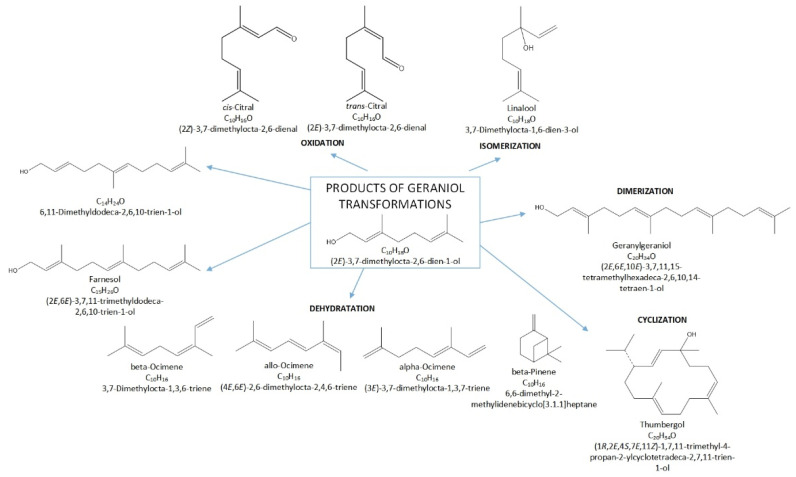
Diagram of the main chemical reactions taking place during the transformations of GA.

Terpenes, including GA, are natural products that can be easily transformed into valuable compounds, which are then used in industrial methods of producing perfumes, various types of aromatic or therapeutic agents [2]. Geraniol undergoes numerous changes in the presence of porous catalysts, hence the process, which was originally to be carried out as the isomerization process of this compound, becomes a very complicated process to describe. For geraniol the following possible transformations can be described: dehydration (products in the form of β-pinene (the equivalent name is beta-pinene) (BP) and ocimene (OC)), oxidation (products in the form of (cis-, trans-) citral (CI)), isomerization (products in the form of linalool (LO) and nerol (NO)), dimerization (geranylgeraniol (GG)), cyclization and fragmentation carbon chain (6,11-dimethyldodeca-2,6,10-trien-1-ol (DC) and thumbergol (TH)) (Figure 2).

The aim of further research on this process should be to find a very efficient heterogeneous catalyst that would permit 1, maximum 3 products obtained with high selectivity in this process.

One of the products of dehydration of geraniol is β-pinene (main product obtained in our work during studies in the presence of diatomite). β-Pinene is an organic compound from the group of bicyclic monoterpenes. Due to its properties, it is used in the cosmetics industry, as a raw material for the production of other fragrances such as: bergaptol, limonene and terpineol. Additionally, it is used as an anti-inflammatory, anti-cancer, expectorant and bronchodilator [17]. It was described that beta-pinene is used in the production of drugs applied in treatment of the liver and kidneys [18] and also it was shown that this compound may be applied as an effective antiallergic agent [19]. The anxiolytic properties of pinene were also confirmed [20]. The potential for pain relief of neuropathic and inflammatory types was investigated using the essential oil of *Ugni myricoides* leaves. Researchers attribute the obtained effects to the presence of pinene [21]. Beta-pinene can be also used as an antibacterial agent [22,23] and this compound may be helpful in the treatment of Alzheimer’s and other neurodegenerative diseases [24].

Other products obtained as a result of the transformation of GA are cymenes-organic chemical compounds belonging to the group of monoterpenes. They are mainly used in the perfume industry [25].

Linalool (isomerization product) belongs to aliphatic unsaturated alcohols. As a fragrance or as linaloolyl acetate, it has found application in the perfume industry. This compound is also used in treatment of leukemia or cervical cancer [26].

Nerol oil is known for its properties supporting the renewal of skin cells, which translates into improvement of its elasticity, maintenance of the appropriate serum level, reduction of wrinkles and scars. It is used in treatment of bacterial skin infections caused by fungi, bacteria or yeasts [27].

Another valuable product of GA transformation is citral (oxidation products). Citrals are used in the perfume and food industry and also in medicine-citrals have antibacterial properties and, moreover, are used in cancer treatment [28].

Thumbergol (one of the main products obtained in our work for studied in the presence of alum) is diterpene monocyclic alcohol used in cancer treatment This compound also shows neuroprotective [29] and antibacterial properties [30,31].

Literature data on the transformation process of GA are not sufficiently described. Yu et al. [32] presented the transformation of GA with the presence of the FeCl_2_ × 6H_2_O as the catalyst. The main products obtained in this process were linalool and α-terpineol. If the process was carried out in acetonitrile with water (5%), linalool yield was 26%, while if the process was carried out in anhydrous acetonitrile, linalool yield was 4%, and α-terpineol yield was 53%. Studies in the presence of mineral showed that the only product was α-terpineol which was formed with less than 10% yield.

Haese et al. [33] presented studies on transformations of pure GA, and in mixture with nerol. The isomerization was carried out at 160 °C and at vacuum. As the catalyst oxodiperoxotungstic acid was used. The main product for pure Ga and Ga in mixture with nerol was linalool.

Srivastava et al. [34] presented the transformation of GA under the influence of gamma radiation, the source of which was ^60^Co. As a result of irradiation of the GA-methanol solution, GA was converted to nerol and linalool, and the conversion of GA reached value of 30%.

Ramishvili et al. [35] and Tsitsishvili et al. [36] transformed GA in the presence of micro- and mesoporous zeolites of the BEA type. The obtained results show that GA was converted mainly to linalool and nerol, as well as to: (2*E*,6*E*)-6,11-dimethyldodeca-2,6,10-triene-1-ol and (*trans, trans*-farnesol-(2*E*,6*E*))-3,7,11-trimethyldodeca-2,6,10-trien-1-ol). During studies the conversion of GA reached 99%.

Fajdek-Bieda et al. [37] carried out the process of transformations of GA in the presence of sepiolite. The main products in the process were: β-pinene, ocimenes, linalool, nerol, citrals and thumbergol. The highest value of selectivity was obtained for linalool (19 mol%) at the GA conversion amounted to 100 mol%.

In the article by Fajdek-Bieda et al. [38] was described the process of GA transformation, which was performed in the presence of clinoptilolite. The main products were DC and TH. Optimal conditions for obtaining of DC and TH were: temperature 140 °C, catalyst content 12.5 wt % and the reaction time of 180 min. At these conditions GA conversion was 98 mol%, and the selectivities of DC and TH were 14 and 47 mol%, respectively.

Natural minerals are very interesting heterogeneous catalysts used in catalytic reactions, mainly because their availability in the form of numerous deposits and relatively low price [39]. Diatomite (SiO_2_·nH_2_O) is a mineral from the group of siliceous sedimentary rocks, consisting mainly of opal and cristobalite. Diatomite can be used as the porous catalyst in many organic syntheses [40,41,42,43,44,45]. In Poland, its deposits occur in the south of Poland, near Krosno [46,47,48]. Diatomite is also used as the filtering agent and as the absorbent for liquid fertilizers, disinfectants and insecticides [49,50,51,52]. Alum (potassium aluminum sulphate dodecahydrate) is a natural mineral with the chemical formula KAl (SO_4_)_2_ · 12 H_2_O) [53]. It occurs in the form of a crystal that is brittle and easily soluble in water [54,55]. This mineral crystallizes in the form of regular octagonal crystals. Due to its properties, it is used for the treatment of fireproof fabrics as well as for clarifying cloudy water [56,57,58,59]. Alum is used in the synthesis of synthetic ethyl alcohol, but scientific literature lacks a large amount of information on the catalytic use of alum. One of the few literature reports shows the use of potash alum as a sustainable heterogeneous catalyst in a one-pot synthesis of highly functionalized pyrrol-2-ones and furan-2-ones [60]. Examples of alum occurrence are: Uzbekistan, Italy—Vesuvius, while in Poland Sandomierz, Międzyzdroje and Lower Silesia [61,62].

Natural minerals are very interesting heterogeneous catalysts used in catalytic reactions, mainly because their availability in the form of numerous deposits and relatively low price [39]. Diatomite (SiO_2_·nH_2_O) is a mineral from the group of siliceous sedimentary rocks, consisting mainly of opal and cristobalite. Diatomite can be used as the porous catalyst in many organic syntheses [40,41,42,43,44,45]. In Poland, its deposits occur in the south of Poland, near Krosno [46,47,48]. Diatomite is also used as the filtering agent and as the absorbent for liquid fertilizers, disinfectants and insecticides [49,50,51,52]. Alum (potassium aluminum sulphate dodecahydrate) is a natural mineral with the chemical formula KAl (SO_4_)_2_ · 12 H_2_O) [53]. It occurs in the form of a crystal that is brittle and easily soluble in water [54,55]. This mineral crystallizes in the form of regular octagonal crystals. Due to its properties, it is used for the treatment of fireproof fabrics as well as for clarifying cloudy water [56,57,58,59]. Alum is used in the synthesis of synthetic ethyl alcohol, but scientific literature lacks a large amount of information on the catalytic use of alum. One of the few literature reports shows the use of potash alum as a sustainable heterogeneous catalyst in a one-pot synthesis of highly functionalized pyrrol-2-ones and furan-2-ones [60]. Examples of alum occurrence are: Uzbekistan, Italy—Vesuvius, while in Poland Sandomierz, Międzyzdroje and Lower Silesia [61,62].

In this work, we investigated the transformations of GA in the presence of natural catalysts in the form of diatomite and alum. The studies tested the influence of temperature, catalyst content, and reaction time on the course of the GA transformation process. The syntheses were carried out under the atmospheric pressure, in the air atmosphere and without the use of any solvent. The absence of solvent is advantageous as this eliminates the possibility of the reaction used solvent with the GA transformation products as well as the need to recover and recycle the solvent to the process. The aim of the study was to compare the catalytic activity of diatomite and alum and to find the most favorable conditions for the transformation of GA on these catalysts. Before catalytic studies with GA the catalysts were described with the following instrumental methods: XRD, SEM/EDX, FTIR and XRF methods. Thanks to the use of these methods a full characterization of their physicochemical properties was prepared.

## 2. Materials and Methods

### 2.1. Raw Materials

The syntheses were performed in the presence of diatomite (100% pure, Nanga, Hobbs, New Mexico) and aluminum potassium alum (100% pure, Nanga, Ufa, Russia) as the catalysts. The organic raw material used in our tests was GA (99% pure, from Acros Organics, Milwaukee, USA). For quantitative analysis which were performed by the gas chromatography method (GC), standards in the form of: citronellol (95% pure, from Sigma Aldrich, Steinheim, Germany), citral (95% pure, Sigma Aldrich, Steinheim, Germany), ocimen (90% pure, from Sigma Aldrich, Milwaukee, WI, USA), beta-pinene (95% pure, from Fluka, Milwaukee, WI, USA), linalool (97% pure, from Acros, Steinheim, Germany), farnesol (96% pure, from Acros, Steinheim, Germany), nerol (97% pure, from Acros, Steinheim, Germany), myrcene (pure technical from Sigma Aldrich, Steinheim, Germany) and geranylgeraniol (85% pure, from Sigma Aldrich, Milwaukee, WI, USA) were used.

### 2.2. Characteristics of Diatomite and Alum

For the characteristc of diatomite and ałum the following method were used:

— X-ray diffractometry (XRD)—Empyrean X-ray diffractometer with Cu Kα radiation source (Malvern Panalytical, Grovewood, UK); analysis of samples in the temperature range of 5–30° in 0.02° steps;
— Specific area (SSA), total pore volume (TPV) and micropore volume (MV)—nitrogen adsorption method at 350 °C using the QUADRASORB evoTM Gas Sorption Surface and Pore Size Analyzer (Quantachrome Instruments, Boynton Beach, FL, USA), prior to analysis samples were degassed at 250 °C for 20 h in atm. N_2_;
— Mapping of elements—scanning electron microscopy (SEM) and EDX surface spectra-SEM apparatus (JEOL company, JSM-6010LA, Tokyo, Japan) with a secondary electron detector;
— Elemental analysis performed with Epsilon3 energy dispersed X-ray fluorescence spectrometer (EDXRF) (Malvern Panalytical, Grovewood, UK);
— FT-IR infrared spectorscopy (Thermo Nicolet 380 apparatus, Malente, Germany)—wavenumber range from 400 to 4000 cm^−1^.

### 2.3. Method of Transformations of Geraniol and Analyses of the Post-Reaction Mixtures

The syntheses were carried out in a glass reactor with a capacity of 25 cm^3^, which was equipped with a reflux condenser and a magnetic stirrer with heating function. The ranges of the studied parameters were as follows: temperature 80–150 °C, catalyst content 5–15 wt %, reaction time from 15 min to 24 h. In order to perform a qualitative and quantitative analyses, the sample of the post-reaction mixture was first centrifuged and then it was dissolved in acetone in the ratio 1:3.

Qualitative analyses were performed using the GC-MS method on a ThermoQuest apparatus with a Voyager detector and a DB-5 column (filled with phenylmethylsiloxanes, 30 m × 0.25 mm × 0.5 mm). Analysis parameters: helium flow 1 mL/min, sample chamber temperature 200 °C, detector temperature 250 °C, oven temperature—isothermally for 2.5 min at 50 °C, then heating at the rate of 10 °C/min to 300 °C. Quantitative analyses were performed with help of Thermo Electron FOCUS chromatograph with FID detector and TR-FAME column (cyanopropylphenyl packed, 30 m × 0.25 mm × 0.25 mm). The analysis parameters were as follows: helium flow 0.7 mL/min, sample chamber temperature 200 °C, detector temperature 250 °C, oven temperature—isothermally for 7 min at 60 °C, then heating at the rate of 15 °C/min to 240 °C. The FID temperature was kept at the level of 250 °C. Examples of GC-MS analyzes in the presence of alum and diatomite are included in the Supplementary Material.

The quantitative analyses of the products were performed using the external and internal standard method. In case of the first method, 8-point calibration curves were performed for each compound in the concentration range of 0–33 wt %. After the chromatographic analyses the mass balances for each synthesis were prepared. The mass balances allowed us to calculate the main functions describing the process:

(1) $$S_{\mathrm{product}/\mathrm{geraniol}}=\frac{\mathrm{Amount}\;\mathrm{of}\;\mathrm{moles}\;\mathrm{of}\;\mathrm{product}}{\mathrm{Amount}\;\mathrm{of}\;\mathrm{moles}\;\mathrm{of}\;\mathrm{geraniol}\;\mathrm{consumed}}\times \;100\%$$

(2) $${\;C}_{\mathrm{geraniol}}=\frac{\mathrm{Amount}\;\mathrm{of}\;\mathrm{moles}\;\mathrm{of}\;\mathrm{geraniol}\;\mathrm{consumed}}{\mathrm{Amount}\;\mathrm{of}\;\mathrm{moles}\;\mathrm{of}\;\mathrm{geraniol}\;\mathrm{introduced}\;\mathrm{into}\;\mathrm{reactor}}\times \;100\%$$

## 3. Results

### 3.1. Morphology of Natural Diatomite and Alum

**Figure 3:**
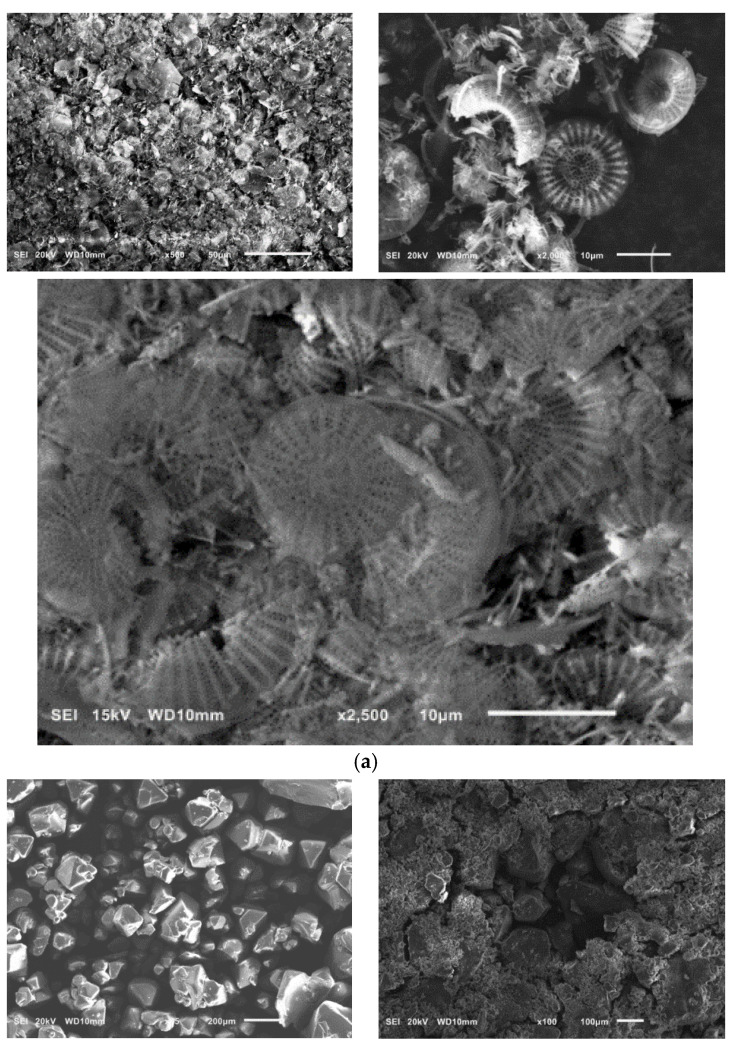

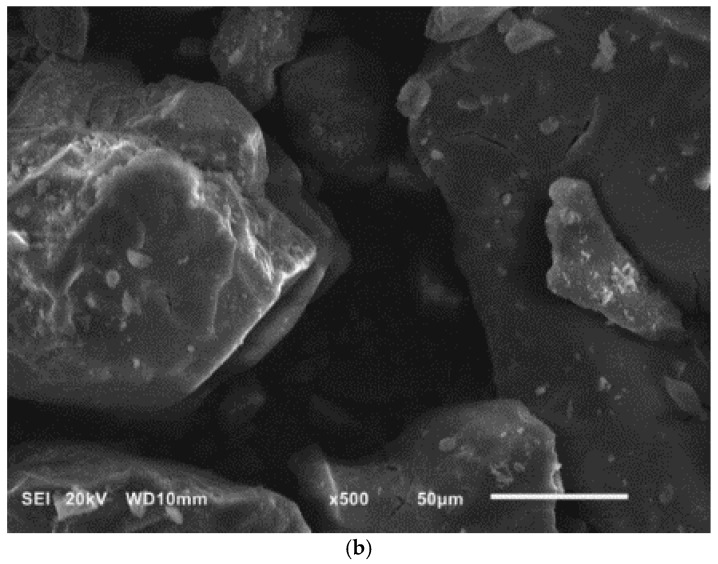
SEM images of diatomite (**a**) and aluminium potassium alum (**b**) (own study).

SEM pictures of natural diatomaceous earth-diatomite are shown in Figure 3a. The photos show the diatom skeleton with an ordered micro- and nanoporous structure [54,55]. Additionally, numerous clusters of diatomaceous shells are visible. The radius of such a diatom is about 15 µm. Diatomite is characterized by a large volume of voids as well as a significant porosity of its structure [63,64,65,66].

Alum-potassium crystals form agglomerates of irregular shape (Figure 3b). Due to the varied shapes of the particles of this mineral, it is characterized by a heterogeneous structure. These observations have also been described by other authors for potassium aluminum alum [67].

**Figure 4:**
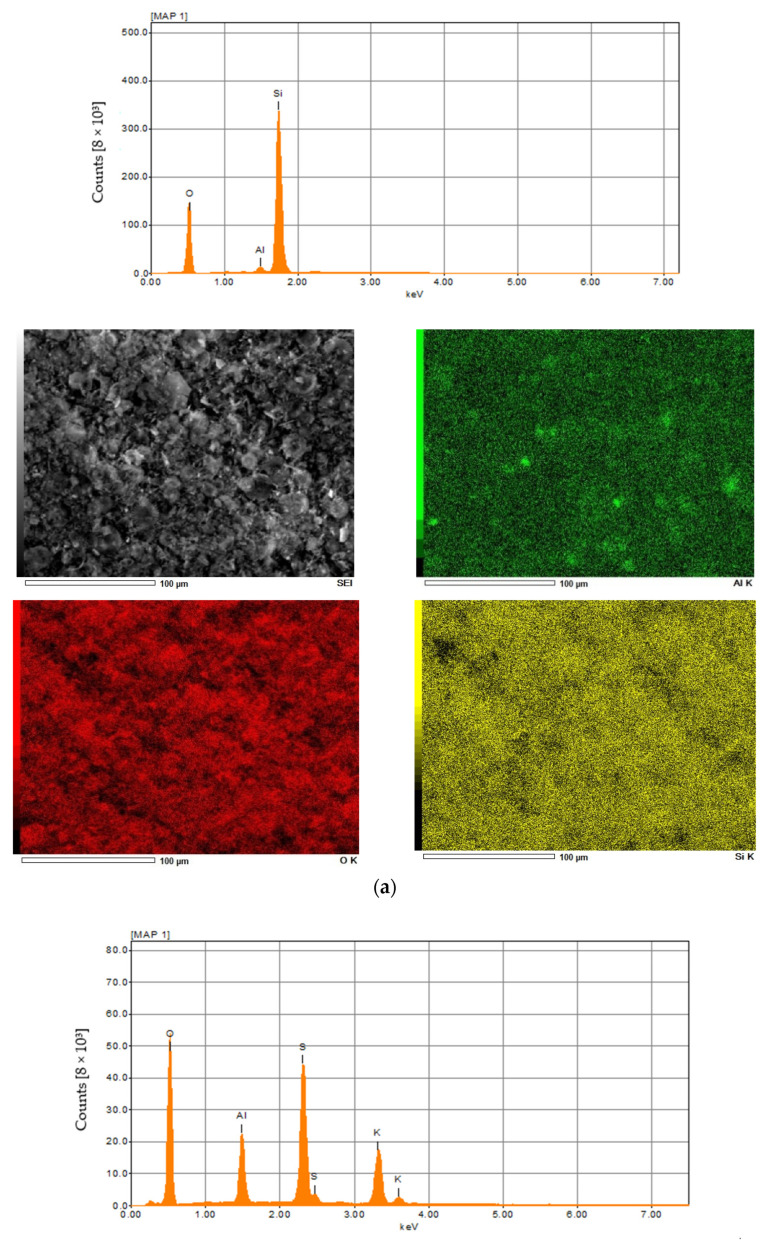

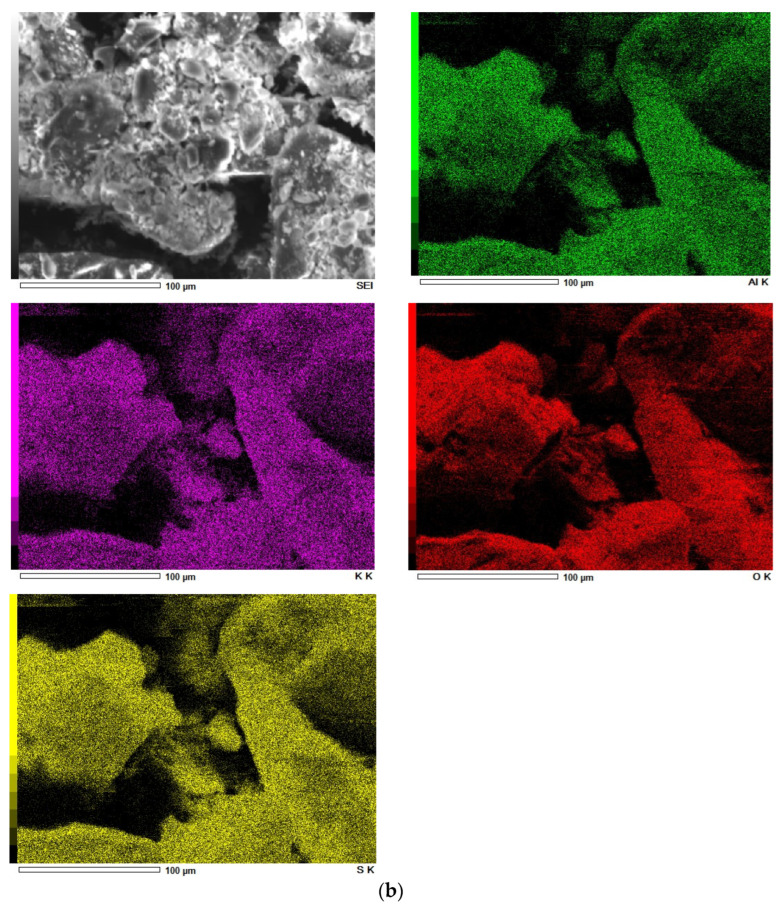
The XRF and EDX spectra of diatomite (**a**) and aluminium potassium alum (**b**).

The results of the elemental analysis of diatomite and aluminum-potassium alum are summarized in Figure 4 and in Table 1 and Table 2. In the case of diatomite, the highest concentration was achieved for oxygen and was 53 wt %. The second most important element was silicon, which concentration was about 46 wt.%, then aluminum in the amount of about 1 wt.% (Figure 4a). In other studies, the oxygen concentration is slightly lower (about 48 wt.%) and additionally there is iron, the presence of which may be caused by the location or small impurities [63]. Elemental analysis in the case of aluminum potassium alum showed that the most important elements found in the structure of this mineral are oxygen in the amount of 63 wt.%, sulfur in the amount of 18 wt.%, potassium in the amount of 11 wt.% and aluminum in the amount of about 8 wt.% (Figure 4b). The obtained results for both catalysts are similar to those obtained by other authors [67].

**Figure 5:**
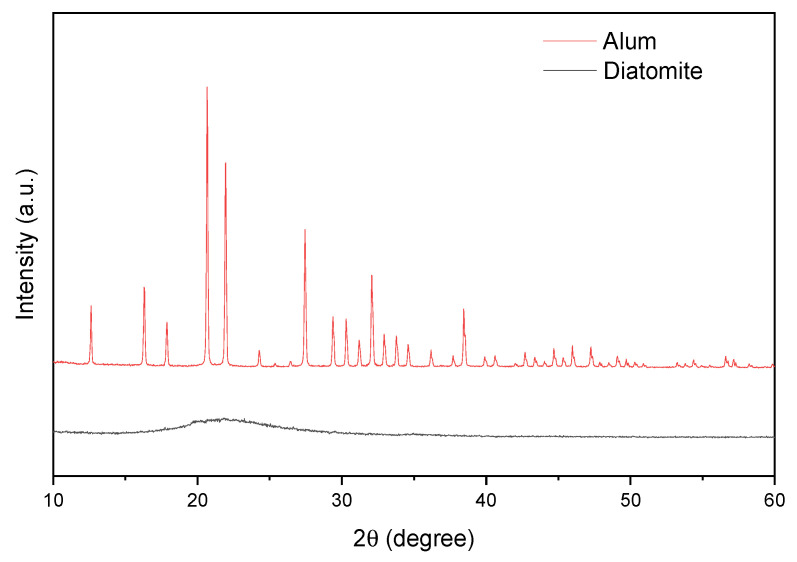
X-ray diffraction data for catalysts.

Figure 5 shows the diffractograms of diatomite and alum. The pattern acquired from diatomite was found identical to that of reported in literature [68]. Broad peak located at 22° (2θ) corresponds to amorphous silica and is characteristic of natural diatomite. The XRD alum spectrum is consistent with the literature (JCPDF 07-0017) and is responsible for the potassium alum [67].

In order to fully characterize the tested materials, their BET surface area and total pore volume were determined—Table 3. Comparing the BET surface area for diatomite with the data from the literature results, the investigated mineral locality has a larger surface area (33.6 m^2^/g) and a larger pore volume (0.12 cm^3^/g) in relation to literature data [69]. The second of the best catalysts is potassium aluminum alum with a very small surface area and pore volume, which are, respectively, 1.1 m^2^/g and 0.006 cm^3^/g.

**Figure 6:**
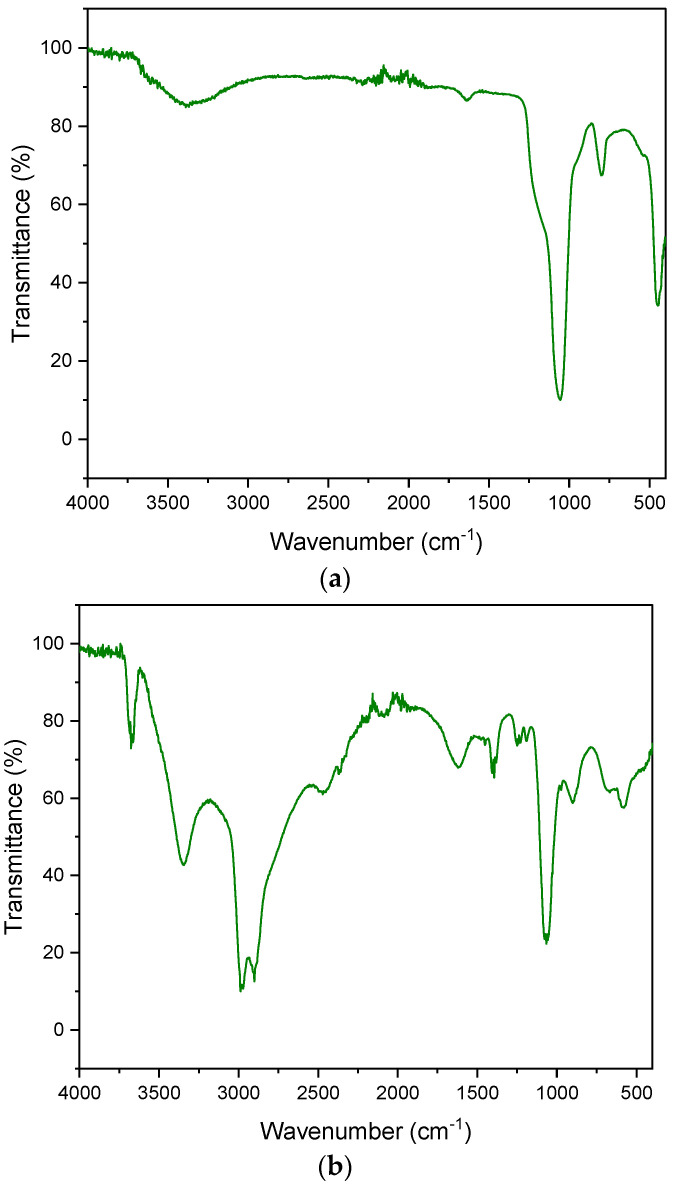
FT-IR spectrum. (**a**) diatomite; (**b**) aluminium potassium alum.

The FT-IR spectrum presented for diatomaceous earth shows characteristic adsorption bands at 3436, 1625, 1094 and 797 cm^−1^ (Figure 6):

— 3436 cm^−1^ confirms the presence of a free silanol group (SiO-H) on the surface of the quantum,
— 1625 cm^−1^ is characterized by the HOH bending vibrations of water,
— 1094 cm^−1^ is responsible for the stretching vibrations of the siloxane group (-Si-O-Si),
— 797 cm^−1^ is characteristic of the vibrations of the SiO-H group.

Figure 6b shows the spectrum of aluminum potassium alum. The bands at the 1195 cm^−1^ and 1077 cm^−1^ wavelengths are responsible for the stretching vibrations of the S = O groups. The bands occurring at the lengths of 933 cm^−1^ and 737 cm^−1^ are the result of stretching vibrations of the S = O group. The peaks that appeared in the vicinity of 750–400 cm^−1^ indicate Al-O vibrations. The characteristic sharp peaks of sulphate (SO_4_^2−^) occur at 468–471 cm^−1^, 603–608 cm^−1^, 657–686 cm^−1^, 1104–1115 cm^−1^ and 1237–1247 cm^−1^. FT-IR spectra are very similar to those presented by other authors [65,66,67].

### 3.2. The Influence of Temperature on GA Transformations

The first analyzed parameter was the process temperature in the range of 80–150 °C. The initial parameters were as follows: catalyst content 5 wt % and a reaction time of 3 h.

Table 4 shows the influence of temperature on the first of the analyzed functions—the conversion of GA. In case of diatomite, this function values increase in the entire tested temperature range from about 82 mol% (80 °C) to about 100 mol% (150 °C). A similar course for this function can be observed for the second catalyst—aluminium potassium alum, for which about 93 mol% conversion of GA was obtained for the temperature of 80 °C and about 100 mol% for 150 °C. The increase in GA conversion with increasing temperature can be explained by the increase in the diffusion rate of GA molecules into the pores, where there are active centers (aluminum atoms) on which the geraniol reactions we are examining take place.

**Figure 7:**
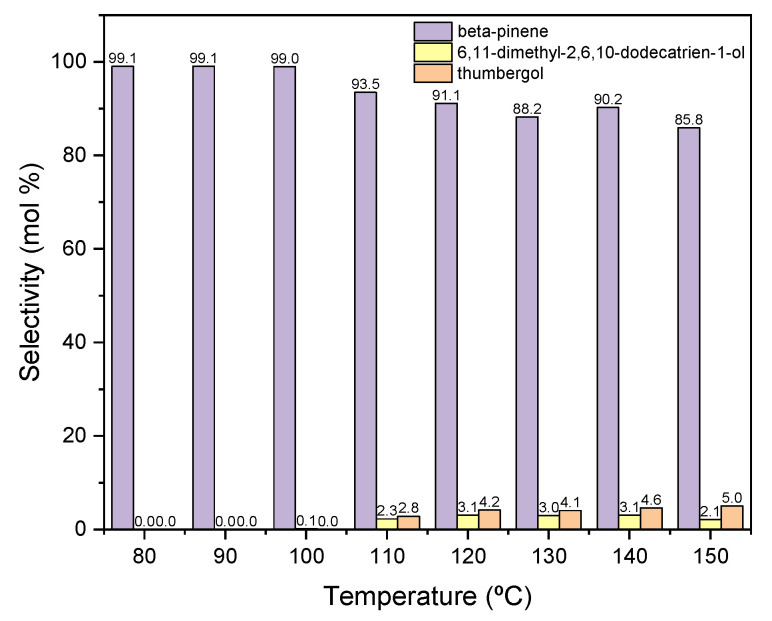
The influence of temperature on the selectivities of the appropriate products over diatomite as the catalyst.

Other functions describing the transformation process are the selectivities of the transformation of GA to the appropriate products of the process. Figure 7 shows the courses of the selectivity for individual products when the process is carried out in the presence of diatomite.

**Figure 8:**
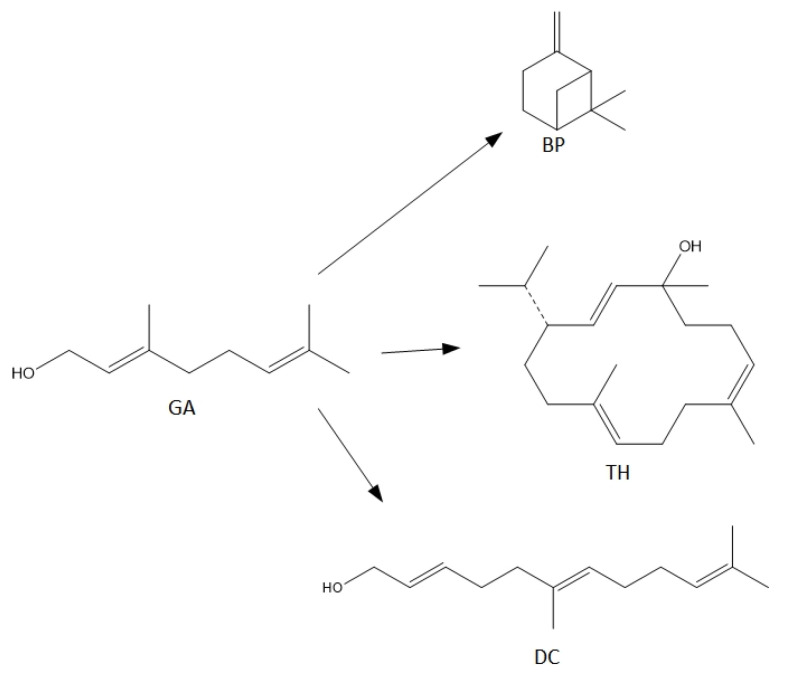
The main reactions describing process of transformations of geraniol in the presence of diatomite.

For the syntheses performed in the presence of this catalyst the main reactions can be described as follows (Figure 8):

When the process is carried out at low temperatures in the range of 80–100 °C, only the dehydration product is formed in the form of BP, with a selectivity of about 100 mol% (Figure 7). As the temperature rises above 110 °C, cyclization product—TH and fragmentation product—DC, the selectivities of which increase to about 3 mol% (for DC) and to about 5 mol% (for TH).

Due to the obtained results, 80 °C was chosen as the most favorable temperature for carrying out the reaction in the presence of diatomite, mainly due to the highest selectivity of the transformation to BP, despite the low conversion of GA.

**Figure 9:**
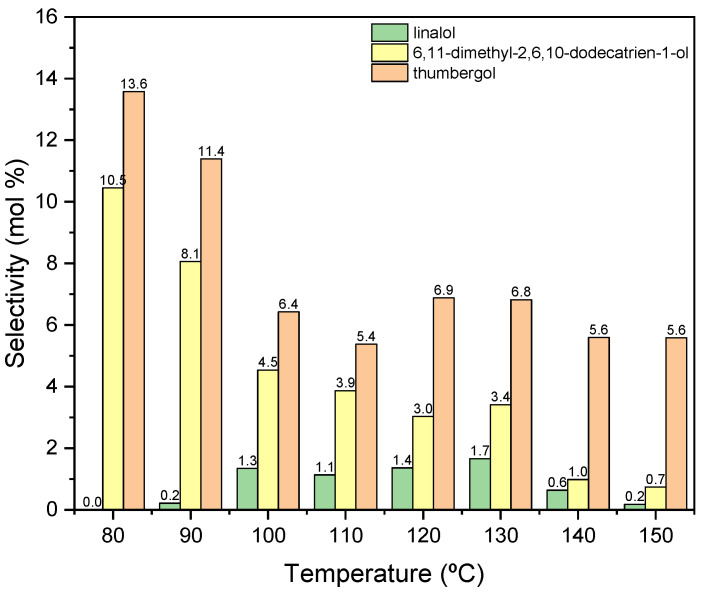
The influence of temperature on the selectivities of the appropriate products over aluminium potassium alum as the catalyst.

The next figure (Figure 9) shows the influence of temperature for the second tested catalyst-alum, on the selectivities of the appropriate products. 

**Figure 10:**
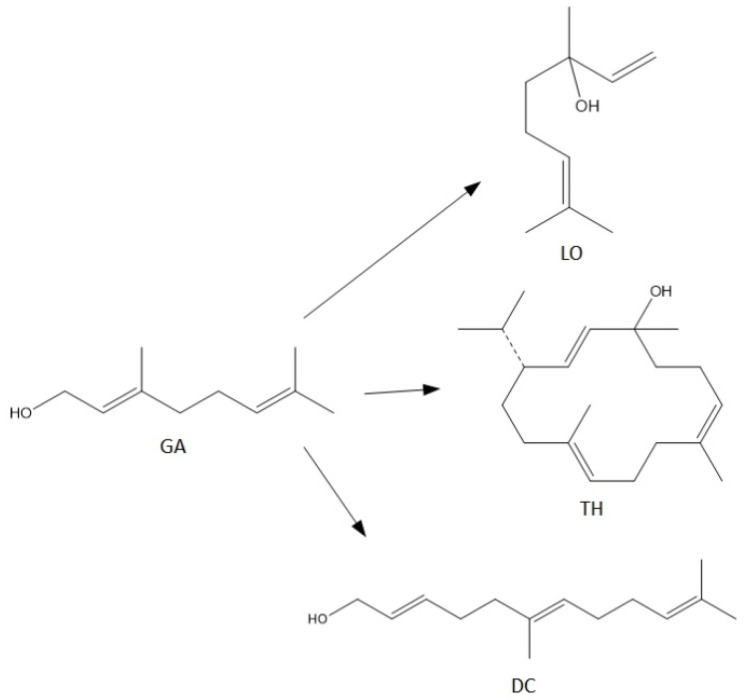
The main reactions describing process of transformations of geraniol in the presence of alum.

For the syntheses performed in the presence of this catalyst the main reactions can be described as follows (Figure 10):

In this case (Figure 9), the obtained products show significantly lower selectivity values in relation to that when the process was carried out in the presence of diatomite. At the initial temperatures of the process, i.e., 80–90 °C, only cyclization (TH) and fragmentation (DC) products are formed: and at these temperatures, the above-mentioned products achieve the highest selectivities: DC about 11 mol% (80 °C) and TH about 14 mol% (80 °C). With increasing the reaction temperature, the selectivity values for these products decrease, and after exceeding 130 °C, DC is transformed into TH, which can be seen in the increase in the selectivity of this product. Moreover, in the range of 100–130 °C, a third product appears-linalool with the selectivity about 2 mol%.

For further research, 80 °C was selected as the most favorable temperature for carrying out the process in the presence of alum, mainly due to the high selectivity values of the resulting products and high conversion of GA.

### 3.3. The Influence of Catalyst Content on GA Transformations

In the next stage, the influence of the catalyst content (diatomite and alum) was investigated. The parameters for the syntheses were as follows: temperature 80 °C (the most favorable temperature for both catalysts were adopted on the basis of the results from the previous research stage) and the reaction time 3 h.

Table 5 shows the effect of the catalyst content on the conversion of GA. Both in the case of the first and the second catalyst, this function follows a similar course. As the catalyst content increases, its values decrease. For diatomite, it is from about 96 mol% (1 wt %) to about 78 mol% (10 wt %), and for aluminium potassium alum from about 96 mol% (1 wt %) to about 90 mol% (10 wt %). This can be explained by the fact that in the presence of a large amount of catalyst, oligomerization and polymerization reactions of the compounds obtained in this work can take place. We do not mark these relations, but as a consequence we acquire a lower GA conversion value.

**Figure 11:**
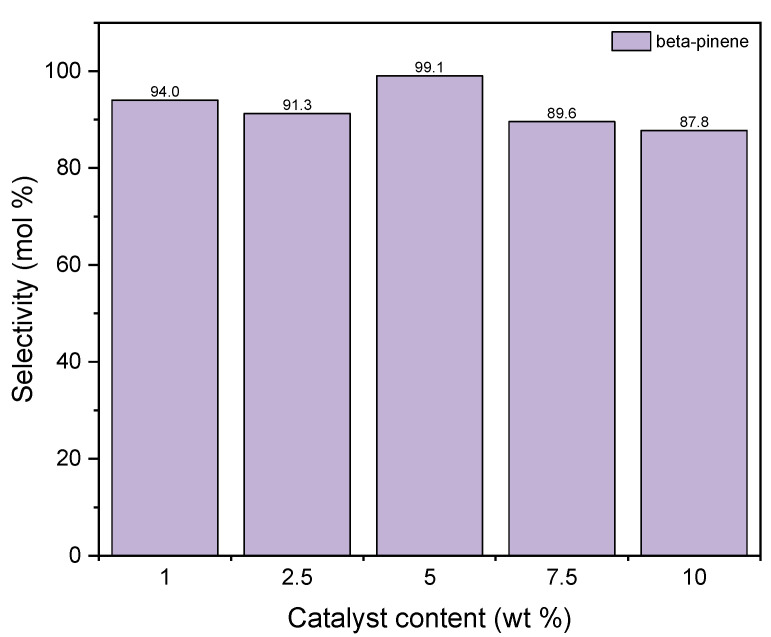
The influence of catalyst content on the selectivities of the appropriate products over diatomite as the catalyst.

Figure 11 shows the changes of the selectivity of transformation to BP when the process is carried out in the presence of diatomite. It is visible that the maximum of this function is achieved for the catalyst content of 5 wt % and amounts to approximately 99 mol%. After this content is increased, the value of selectivity of transformation to BP decreases to 88 mol%. The high activity of diatomite in the formation of beta-pinene can be explained on the basis of the BET method. Compared to alum, diatomite has a well-developed surface and probably the preference for beta-pinene formation over other products is due to the fact that its formation reaction takes place inside the pores where the active centers of aluminum are present. Therefore, we are dealing here with the shape-selective action of diatomite. In the pores of the diatomite, the geraniol molecule transforms from a linear molecule into a monocyclic one as a result of hydration, which takes place on the active centers of Al. This indicates that the cyclic structure of 10 carbon atoms is more preferable in the pores of the diatomite than linear. Low selectivities for the formation of DC and TH compounds on the diatomite may indicate that these compounds are formed mainly on the catalyst surface and their formation requires an increased content of aluminum centers on the surface, and such a situation occurs in the case of alum, where an increase in the selectivity of formation is observed these two products.

**Figure 12:**
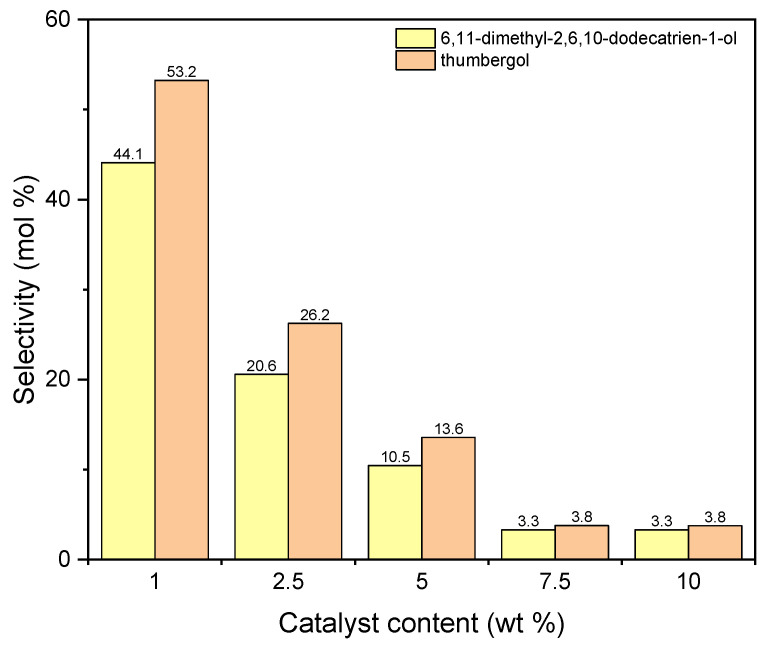
The influence of catalyst content on the selectivities of the appropriate products over aluminium potassium alum as the catalyst.

The next graph (Figure 12) shows the selectivities of main two products obtained during carrying out the process with the use of aluminium potassium alum. The selectivity values for both compounds decrease with increasing in catalyst content. For DC, from 44 mol% (1 wt %) to about 3 mol% (5 wt %), and for TH, from 53 mol% (1 wt %) to about 4 mol% (5 wt %).

Products marked as DC and TH (a chain compound with the number of C atoms equal to 14 and a cyclic compound with the number of C atoms equal to 20) are actually formed only on a catalyst that does not have a developed specific surface area, but it is characterized by a much higher content of aluminum (7.56 wt %) than diatomite. Taking into account the lack of developed specific surface, it can be assumed that the reaction with alum as a catalyst takes place on its surface. A large accumulation of Al active centers on the alum surface can lead to fragmentation of geraniol molecules, and the fragments formed are attached to geraniol molecules or also to dimerization and cyclization, which leads to compounds containing 20 carbon atoms. The high selectivity of the transformation towards thumbergol may indicate that the formation of a cyclic structure is more privileged than the formation of a linear structure (geranylgeraniol), which is not detected among the products.

Taking into account the results obtained for both catalysts, 1 wt % was chosen as the most favorable content of catalyst in both cases.

### 3.4. The Influence of Reaction Time on GA Transformations

The effect of the reaction time was tested in the range from 15 min to 24 h. The remaining parameters, such as temperature and catalyst content, corresponded to the values previously determined as the most favorable for each of the catalysts.

Table 6 shows the changes of GA conversion as function of reaction time. In case of diatomite, the values of the conversion of GA increase from 15 min (92 mol%) to 180 min (96 mol%), and then after prolongation the reaction time conversion of GA decrease to the value of about 80 mol% and remain at this constant level to the end of tests. The GA conversion values, when alum is used, increase up to 180 min (to 96 mol%) and then slightly decrease and for the reaction time 1440 min it amounts to 89 mol%.

**Figure 13:**
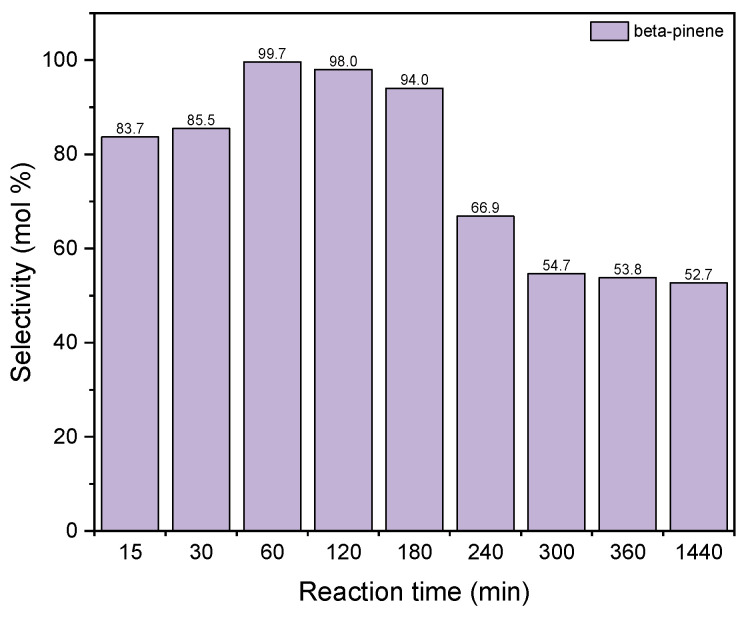
The influence of reaction time on the selectivities of the appropriate product over diatomite as the catalyst.

Figure 13 shows the changes in the values of the selectivity of the transformation to BP when diatomite was used as the catalyst for the process of GA transformations.

The selectivity of transformation to BP increases for the reaction time to 60 min (selectivity value amounts to 100 mol%) and then gradually decreases to about 63 mol% for the reaction time of 1440 min. The reaction time 60 min was chosen as the most preferred time for this catalyst.

**Figure 14:**
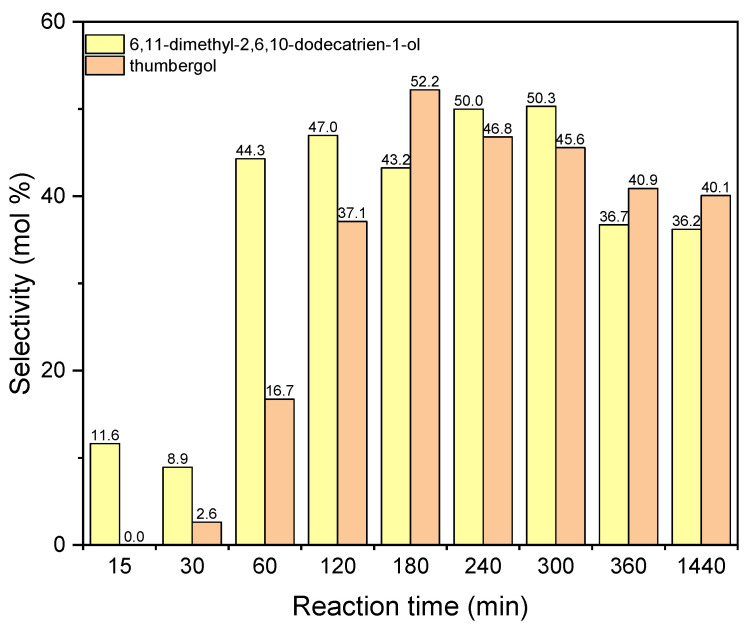
The influence of reaction time on the selectivities of the appropriate products over aluminium potassium alum as the catalyst.

The next figure concerns the studies with aluminium potassium alum and shows the changes in selectivities of DC and TH (Figure 14). The values of selectivities for both compounds increase for the reaction time 180–240 min. For the reaction time 240 min the selectivity of DC is about 50 mol%, and for thumbergol it selectivity amounts 52 mol% for the reaction time 180 min. After the reaction time 180–240 min, the values of selectivity of these two compounds slightly decrease: for DC to about 36 mol% and for TH to about 40 mol%.

Taking into account the results obtained for both catalysts, the following was selected as the most favorable reaction time: 1 h (for diatomite) and for 3 h (for alum).

## 4. Discussion

The conducted studies have shown that both diatomite and potassium aluminum alum are the active catalysts in the process of GA transformations and that all tested parameters (temperature, amount of the catalyst and reaction time) have influence on the course of GA transformations and values of GA conversion and selectivities of the main products. The conducted research shows that the most favorable conditions of the GA transformation process were obtained: at the temperature of 80 °C (for both tested catalysts), with a catalyst content of 1 wt % (for both tested catalysts) and for 1 h (for diatomite) and for 3 h (for alum). The obtained conditions permit the obtainment of reaction products, mainly BP, DC and TH with the highest possible selectivity (99% mol, 44% mol and 53% mol), with a high level of GA conversion (in the range of 96–98% mol). The use of both higher temperature and higher catalyst content and longer reaction times can give rise to undesirable reaction products. Due to the more developed surface, diatomite catalyses the formation of beta-pinene (a product with a smaller molecule) in its pores. On the other hand, the reactions occurring on alum are catalyzed by active centers located on its surface, which allows to obtain, as a result of the transformation of geraniol and small-molecule products formed during its transformation, products with larger particles. The mechanism of changes in geraniol on diatomite and alum, however, requires further detailed research. It is interesting, however, that replacing the catalyst with diatomite allows to obtain a low molecular weight product, which is beta-pinene, using the shape-selective action of the pores present in the diatomite.

Comparing the results presented in our previous publications, which described the process of transformation of GA with the use of sepiolite (a mineral from the silicate group, classified as clay minerals) [29] and clinoptilolite (a mineral from the group of silicates, included in the group of zeolites) [30], with these described in this paper, it was observed that the previously tested catalysts required longer reaction time, a much higher catalyst content and higher temperature to obtain high values of selectivity of the transformation to the appropriate products and the conversion of GA. In general, the presented studies showed that increasing in the temperature or extending the reaction time causes a decrease in the value of the selectivity of the formation of compounds that most likely decompose because their structure is not stable.

Undoubtedly, the advantage of the proposed method of GA transformation is the lack of solvent in the reaction medium. Its presence could cause additional reactions with GA and its transformation products, which would increase the number of products produced.

## Figures and Tables

**Table 1:** EDX results—diatomite.

Element	Mass, %	Atom, %
O	53.07	66.48
Al	1.16	0.86
Si	45.77	32.66

**Table 2:** EDX results—aluminium potassium alum.

Element	Mass, %	Atom, %
O	63.30	77.83
Al	7.56	5.51
S	18.17	11.15
K	10.97	5.52

**Table 3:** Properties surfaces of the tested materials.

Catalyst	BET, m^2^/g	V_por_, cm^3^/g
Diatomite	33.6	0.1233
Aluminum potassium alum	1.1	0.0057

**Table 4:** The influence of temperature on the conversion of GA.

Conversion of Geraniol, mol%	Temperature, °C							
	80	90	100	110	120	130	140	150
Diatomite	81.63	79.71	81.17	91.05	94.86	95.86	96.11	99.56
Aluminium potassium alum	92.59	95.15	97.35	97.58	99.54	99.52	99.78	99.82

**Table 5:** The influence of the catalyst concentration on the GA conversion.

Conversion of Geraniol, mol%	Catalyst Concentration, wt %				
	1.0	2.5	5.0	7.5	10.0
Diatomite	96.08	95.76	81.63	79.63	77.22
Aluminium potassium alum	95.58	93.35	92.59	91.00	90.32

**Table 6:** The influence of the reaction time on the conversion of GA.

Conversion of Geraniol, mol%	Reaction Time, min								
	15	30	60	120	180	240	300	360	1440
Diatomite	92.29	91.96	98.87	96.28	96.08	79.51	79.61	80.83	80.08
Aluminium potassium alum	75.10	95.49	82.87	84.34	95.66	86.83	85.91	87.76	88.71

## Data Availability

Not applicable.

## Supplementary Materials

The following supporting information can be downloaded at: https://www.mdpi.com/article/10.3390/ma15072449/s1, Figure S1: Synthesis geraniol-diatomite; Figure S2: Synthesis geraniol-alum.
