# Supplementary material for: Conversion of Geraniol into Useful Value-Added Products in the Presence of Catalysts of Natural Origin: Diatomite and Alum
Source: Materials (Basel). 2022 Mar 26;15(7):2449. doi: 10.3390/ma15072449 (PMC9000025; doi:10.3390/ma15072449)
Supplement: Supplementary file 1 [file materials-15-02449-s001.zip › materials-1591751-supplementary.pdf]

Article

Conversion of Geraniol into Useful Value-Added Products in the Presence of Catalysts of Natural Origin: Diatomite and Alum

Anna Fajdek-Bieda [1], Agnieszka Wróblewska [2,*], Piotr Miądlicki [2] and Anna Konstanciak [1,*]

1 Technical Department, Jacob of Paradies University, Chopina 52, 66-400 Gorzow Wielkopolski, Poland; abieda@ajp.edu.pl
2 Faculty of Chemical Technology and Engineering, Department of Catalytic and Sorbent Materials Engineering, West Pomeranian University of Technology in Szczecin, Piastów Ave. 42, 71-065 Szczecin, Poland; piotr.miadlicki@zut.edu.pl
* Correspondence: agnieszka.wroblewska@zut.edu.pl (A.W.); akonstanciak@ajp.edu.pl (A.K.)

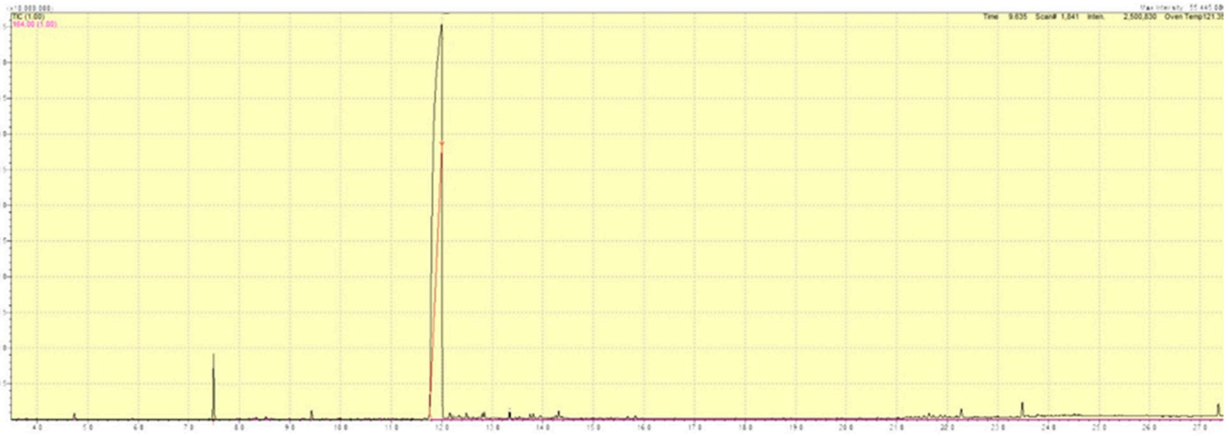

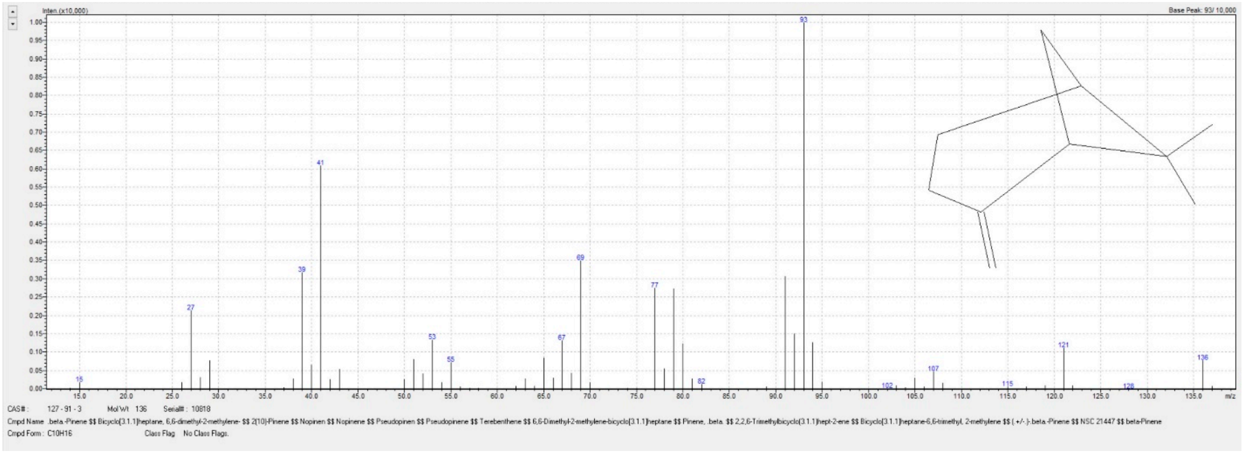

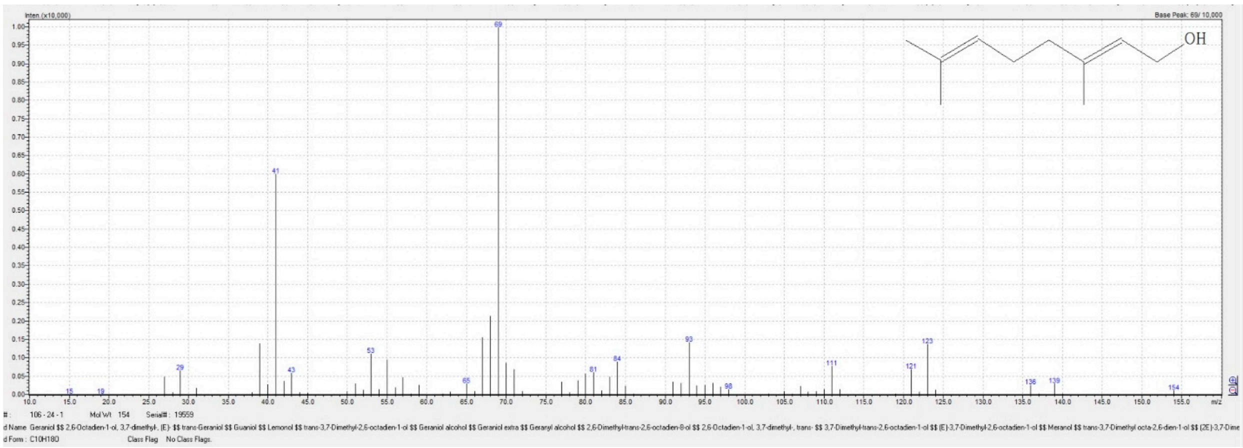

Figure S1. Synthesis geraniol-diatomite.

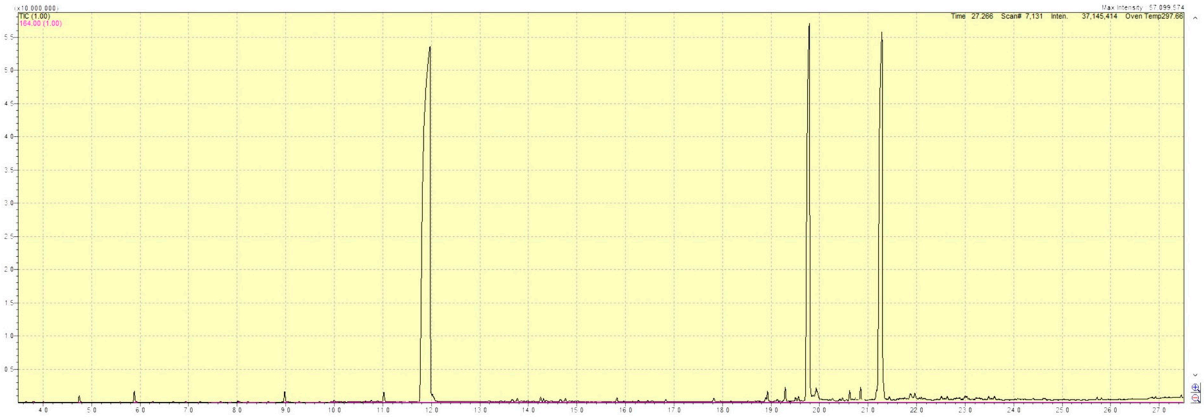

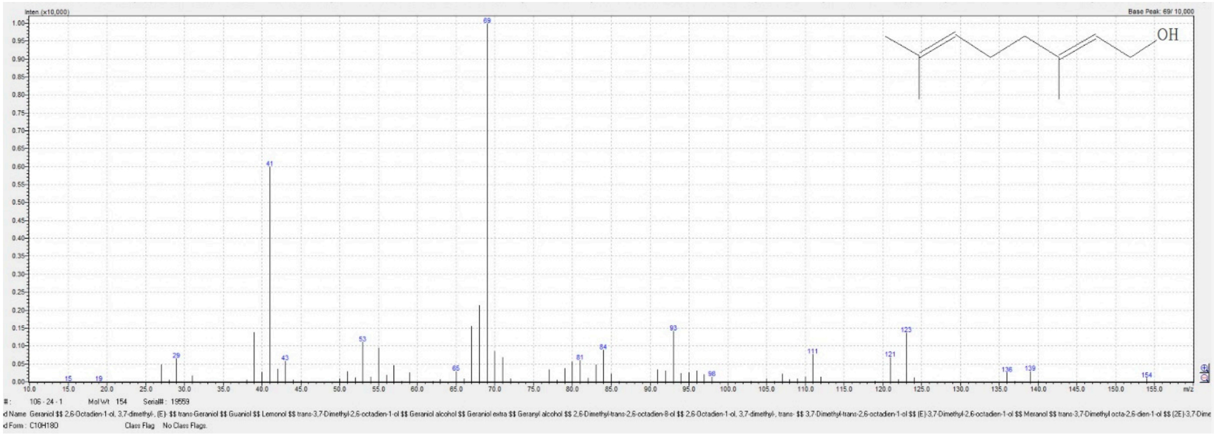

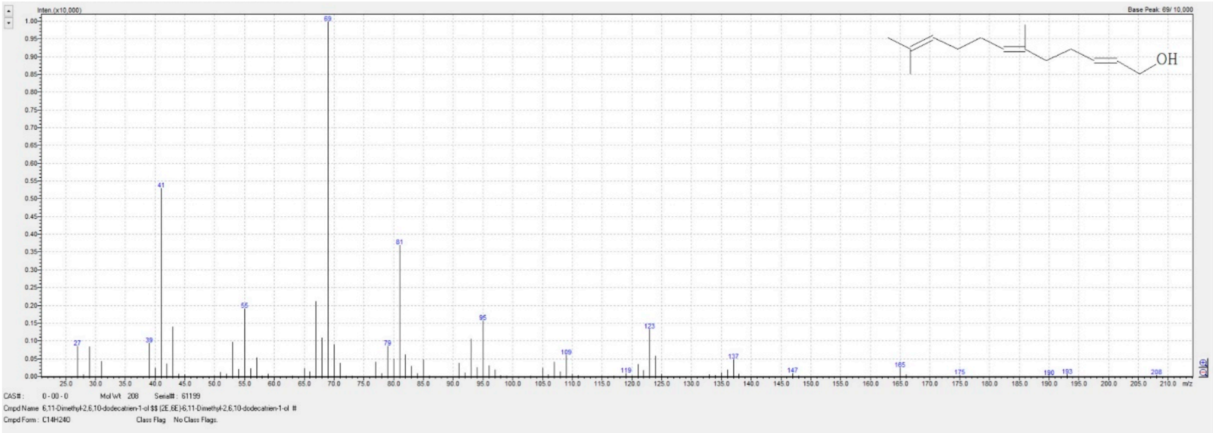

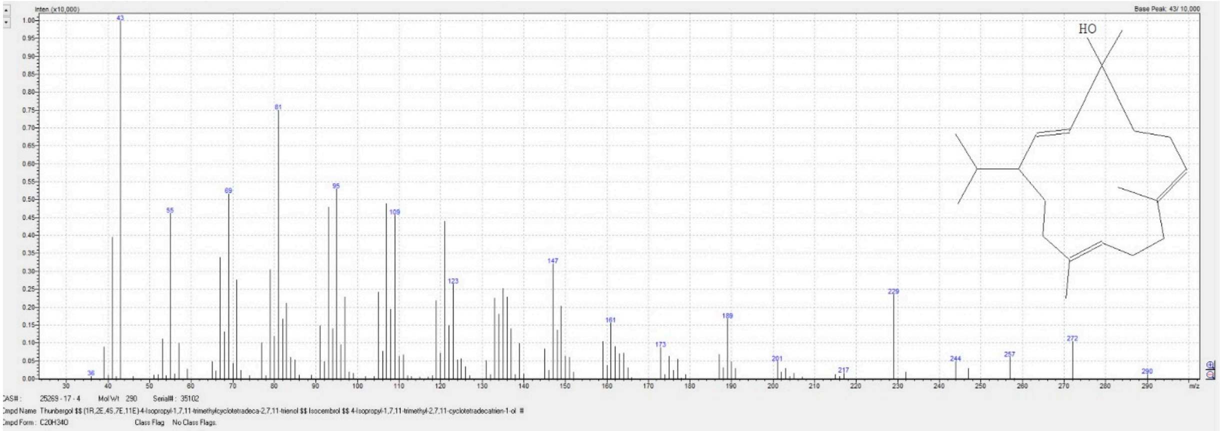

Figure S2. Synthesis geraniol-alum.
